# Draft Genome Sequence Analysis of a *Pseudomonas putida* W15Oct28 Strain with Antagonistic Activity to Gram-Positive and *Pseudomonas* sp. Pathogens

**DOI:** 10.1371/journal.pone.0110038

**Published:** 2014-11-04

**Authors:** Lumeng Ye, Falk Hildebrand, Jozef Dingemans, Steven Ballet, George Laus, Sandra Matthijs, Roeland Berendsen, Pierre Cornelis

**Affiliations:** 1 Department of Bioengineering Sciences, Research group Microbiology, Vrije Universiteit Brussel and VIB Structural Biology Brussels, Brussels, Belgium; 2 Chemistry Department, Vrije Universiteit Brussel, Pleinlaan 2, 1050 Brussels, Belgium; 3 Institut de Recherches Microbiologiques - Wiame, Campus du CERIA, Brussels, Belgium; 4 Plant-Microbe Interactions, Utrecht University, Utrecht, The Netherlands; Charité-University Medicine Berlin, Germany

## Abstract

*Pseudomonas putida* is a member of the fluorescent pseudomonads known to produce the yellow-green fluorescent pyoverdine siderophore. *P. putida* W15Oct28, isolated from a stream in Brussels, was found to produce compound(s) with antimicrobial activity against the opportunistic pathogens *Staphylococcus aureus*, *Pseudomonas aeruginosa*, and the plant pathogen *Pseudomonas syringae*, an unusual characteristic for *P. putida*. The active compound production only occurred in media with low iron content and without organic nitrogen sources. Transposon mutants which lost their antimicrobial activity had the majority of insertions in genes involved in the biosynthesis of pyoverdine, although purified pyoverdine was not responsible for the antagonism. Separation of compounds present in culture supernatants revealed the presence of two fractions containing highly hydrophobic molecules active against *P. aeruginosa*. Analysis of the draft genome confirmed the presence of putisolvin biosynthesis genes and the corresponding lipopeptides were found to contribute to the antimicrobial activity. One cluster of ten genes was detected, comprising a NAD-dependent epimerase, an acetylornithine aminotransferase, an acyl CoA dehydrogenase, a short chain dehydrogenase, a fatty acid desaturase and three genes for a RND efflux pump. *P. putida* W15Oct28 genome also contains 56 genes encoding TonB-dependent receptors, conferring a high capacity to utilize pyoverdines from other pseudomonads. One unique feature of W15Oct28 is also the presence of different secretion systems including a full set of genes for type IV secretion, and several genes for type VI secretion and their VgrG effectors.

## Introduction

*Pseudomonas putida* is a gram-negative rod-shaped γ-proteobacterium found throughout various environments. *P. putida* strains show a diverse spectrum of metabolic activities, including their ability to tolerate heavy metals and to degrade organic solvent, which enables them to survive in highly polluted environments. *P. putida* strains are also known to interact with the rhizosphere and for their plant-growth promoting activities [1]–[7]. Bacteria belonging to the *Pseudomonas* genus produce different bioactive secondary metabolites, but their exploitation is not as developed compared to the situation in Gram-positive bacteria such as *Bacillus* sp. and *Streptomyces* sp. strains [8]. Nowadays, with the advent of the next generation sequencing methods, together with the increased accuracy of gene annotations, new avenues are open for the discovery of secondary metabolite genes clusters in order to gather more information about the different molecules produced and their biological activity. A recent example is the identification, next to the already described antimicrobial compounds pyrolnitrin, pyoluteorin and phloroglucinol, of rhizoxin analogs and orfamides from the well-studied plant-promoting rhizobacterium *P. protegens* Pf-5 (previously named *P. fluorescens* Pf5) through genome-mining [9]. The majority of secondary metabolites with a biological acitivity have so far been described in different strains belonging to the *P. fluorescens* group (although few of these have been renamed, such as *P. protegens* and *P*. *brassicacearum*) [10], but not much is known about the production of bioactive compounds by *P. putida*. *Pseudomonas putida* W15Oct28 was isolated from the Woluwe River, Belgium [11]. This strain showed some unique phenotypic characters such as the production of a new pyoverdine siderophore with a large peptide chain [12] and a capacity to produce compound(s) with anti-microbial activity against *P. aeruginosa* and *Staphylococcus aureus*. Consequently, its potential to produce new secondary metabolites drew our attention, which motivated us to acquire its whole genome sequence by Illumina Miseq technology. In this study, we focus on the secondary metabolite biosynthesis pathways of *P. putida* W15Oct28, which includes pyoverdine, putisolvins biosurfactants, and a novel antimicrobial molecule with broad spectrum inhibitory activity (against *P. aeruginosa*, *P. syringae*, *P. entomophila*, and *S. aureus*, including MRSA), and a partial safracin biosynthetic gene cluster. In addition, we found that this strain presents an extensive repertoire of iron uptake systems with 56 TonB-dependent receptors. W15Oct28 is also unique among pseudomonads because it has a full set of genes for a type IVb (Dot/Icm) secretion system, and several loci for type VI secretion together with five VgrG effector proteins.

## Experimental Procedures

### Bacterial strains and cultivation

*P. putida* W15Oct28 was isolated and purified from samples of the Woluwe river surface water in the frame of a project funded by the Belgian Federal Government who granted us the right to isolate bacteria from the Woluwe and the Senne rivers [11]. With the exception of *P. aeruginosa*, which were cultivated at 37°C, all the other *Pseudomonas* sp. strains were grown at 28°C. *Staphylococcus aureus* strains were cultivated at 37°C. All bacteria were grown in solid or liquid LB medium, except for the production of secondary metabolites (mentioned below). The list of strains and plasmids used in this study, as well as the primers list is presented in **Table S1 in file S1**.

### Secondary metabolites production and purification

For pyoverdine production, *P. putida* W15Oct28 was grown at 28°C in 1 l of iron-poor CAA medium (Bacto Casamino Acid, BD, 5g l^−1^; K_2_HPO_4_ 1.18 g l^−1^; MgSO_4_ • 7H_2_O 0.25 g l^−1^) in 2 l Erlenmeyer flasks, at a shaking speed of 160 rpm for 48 hours. Bacterial cells were removed by centrifugation at 7,000g during 15 min. After filtration the supernatant was passed on a C-18 column that was activated with methanol and washed with distilled water. Elution was done with acetonitrile/H_2_O (70/30%). Samples were lyophilized after most of the acetonitrile was evaporated [13]. The pyoverdines from different *Pseudomonas* sp. strains used for growth stimulation tests were purified by the same protocol, but using smaller scale cultures (20 ml).

For putisolvins and antimicrobial molecules, *P. putida* W15Oct28 was grown at 28°C in 1 l of iron poor M9 minimal medium (12.8 g of Na_2_HPO_4_, 3.0 g of KH_2_PO_4_, 0.5 g of NaCl, 1.0 g of NH_4_NO_3_, 100 µl of 1M CaCl_2_, 2 ml of 1M MgSO_4_, 10 ml of 20% W/V glucose, for 1 liter, pH 7.0) in a 2 l Erlenmeyer flasks, at a shaking speed of 160 rpm for 48 hours. After 48 hours of culture, another 10 ml of 20% W/V glucose was supplemented to the culture again which was left at 4–8°C for 5 more days. Bacterial cells were separated by centrifugation at 7,000 g during 15 min. The supernatant was extracted by 40% volume of ethyl acetate for the extraction of the antimicrobial molecule. Cells were mixed with 30 ml of ethyl acetate and sonicated for 5 minutes. This extraction contained most of the putisolvins and a partial fraction of the antimicrobial molecule(s).

### DNA extraction and whole genome sequencing

Genomic DNA of W15Oct28 was extracted by Puregen Yeast/Bact Kit B (Qiagen, Cat. No. 158567). Four genomic DNA extraction samples were combined, further purified and concentrated by the DNA Clean & Concentrator Kit (ZYMO research, Cat. No. D4003S). The genome of *P. putida* W15Oct28 was sequenced at the VIB nucleomics core using the Illumina Miseq system. The library was constructed by the Nextera kit, yielding reads lengths of 150 bp paired end.

### Genome assembly, annotation and analysis

The final genome coverage was about 62 times and the quality filtered sequences from the MiSeq run were *de novo* assembled using Velvet version 1.2.08 [14]. 138 contigs were further combined in scaffolds using SSPACE basic version 2.0, with 99 contigs representing the draft genome. The draft genome was uploaded and annotated by using the RAST website [15]. Each contig was uploaded to antiSMASH to detect the pyoverdine and other secondary metabolites biosynthetic genes clusters [16], [17]. To predict the substrate of the different adenylation domains, antiSMASH gives 4 predictions based on the combination of NRPSPredictor2 SVM, Stachelhaus code, Minowa, and consensus. When the 4 predictions gave identical results, then the prediction was accepted and considered as valid. When the 4 predictions were different, the amino acids sequences of the adenylation domain were further analyzed by PKS-NRPS to compare with the non-ribosomal Stachelhaus code [18]. The circulated draft genome figure and whole genome comparison with *P. putida* GB-1, BIRD-1, and NBRC 14164 were done by CGView [19]. Genomic islands were searched for by submitting the draft genome to Islandviewer [20], and conserved IS elements were identified by IS Finder database (https://www-is.biotoul.fr/). The draft genome of *P. putida* W15Oct28 was examined by CRISPRFinder to detect the presence of CRISPR elements, which confer immunity against incoming DNA, including bacteriophages [21].

### Random transposon mutagenesis and in-frame gene knock-out deletion

Random mutagenesis library was constructed by conjugating the *E. coli* strain containing the plasposon pTn*Mod*OTc [22] into wild type strain W15Oct28 via biparental mating. Mutants were harvested after antibiotic selection and were selected by functional screening. Information of the insertion flanking regions was acquired by sequencing the plasmid which was rescued from mutants genomes [22] with PITC-F/R primers. In frame genes deletions were facilitated by a yeast recombination method as described by Shanks *et al*. [23]. In summary, PCR primers to amplify upstream and downstream DNA fragments were designed around the region to delete. The two fragments were separately amplified, and mixed with double-digested (*Eco*RI and *Bam*HI) shuttle vector pMQ30, single-stranded carrier DNA (deoxyribonucleic acid sodium salt type III from salmon testes, Sigma, D1626), and engineered yeast strain *S. cerevisiae* InvSc1 in Lazy Bone solution (40% PEG 4000, 0.1 M LiAc, 10 mM Tris-HCl, pH 7.5, 1mM EDTA). After incubation at room temperature overnight, the solution was subjected to 42°C, 12 min for heat shock. In the end this transformation product was washed with TE buffer to remove the polyethylene glycol (PEG 4,000), re-suspended in 600 µl of TE buffer before plating on SD-Ura medium (MP biomedicals, 4813065) for selection. The correctly constructed deletion vector was transferred to the recipient strain via conjugation using a donor *E. coli* strain. The merodiploids were selected by antibiotic resistance (Gm) and confirmed by colony PCR. The shuttle vector was removed by culturing the merodiploids in LB+10% sucrose, and positive mutants were selected by PCR screening.

### High-performance liquid chromatography (HPCL) and mass spectrometry (MS)

HPCL analysis was carried out with an Agilent 1100 Series HPCL System with auto-sampler, degaser UV detector and a thermostated column compartment with an operating temperature range from ambient to 105°C. Preparative-scale purification was done using a Gilson 712 semi-preparative HPCL system with a 322 pump, an UV-VIS 156 detector, a manual injector and 206 Fraction Collector. ESI-LC/MS was done by means of a Waters 600E HPCL Pump, a Waters 2487 Dual Absorbance Wavelength Detector and a Fisons VG II Quattro Mass Spectrometer (ESP ionisation). Operating temperature is from ambient to 80°C. The mobile phase consists of a water/acetonitrile/TFA mixture with a gradient going from a mixture water/AcN (97∶3) containing 0.1% TFA to a mixture water/AcN (0∶100) containing 0.1% TFA in 30 min followed by 10 min isocratic run at these conditions, and with a flow rate of 20 ml min^−1^. High resolution mass spectrometry and collision-induced dissociation tandem fragmentation MS were done on a QTof Micro mass spectrometry (positive ion mode). Under standard measurement conditions the sample was dissolved in CH_3_CN/H_2_O (1∶1) containing 0.1% TFA. GC/MS spectra were recorded on a Trace MS Plus (Thermo). Separation was done on a J&W Scientific DBxXLB (30 m, 0.25 mm ID, 0.25 µm film thickness).

### Antagonism tests

Wild-type and plasposon mutants W15Oct28 strains were pre-cultured in 3 ml of LB till and OD of 0.8 was reached. Then 10 µl of each culture was spotted on M9 minimal medium plates. After overnight cultivation, the producing strain was killed by exposure to UV for 2 minutes. Then soft agar containing the different indicator strains (10^5^–10^6^ CFU) was overlaid on the plates. Results were observed after 18 hours of cultivation at the appropriate temperature. To detect the antimicrobial activity of pre-HPCL isolated fractions, each fraction was dissolved in 50% methanol and 10 µl spotted on the plate with indicator strain overlay.

### Pyoverdine utilization test

Pyoverdine cross feeding test was done on a W15Oct28 pyoverdine null mutant (7G11, transposon inserted in NRPS) on CAA +600µM 2, 2′- dipyridyl. Each type of pyoverdine was added as 10 µl of 8 mM pyoverdine on a paper filter. After incubation at 28°C for 48 hours, the presence of a growth stimulation zone surrounding the filter was considered as positive. Wild type strain and 7G11 were grow on the same medium without any pyoverdine supplement as positive and negative control, respectively.

### Phylogenic analysis of *Pseudomonas* sp. strain and clustering of TonB-dependent receptors

Sequence alignments and trees were generated using CLC Main Workbench 6.7.2 (CLC bio, Aarhus, Denmark). Phylogenic analysis of W15Oct28 was done according to the multilocus sequence analysis (MLSA) of four concatenated housekeeping genes method [10]. Average Nucleotide Identity based on BLAST (ANIb) values was calculated using Jspecies [24]. Clustering of TonB-dependent receptors was done by comparing the amino acid sequences of W15Oct28 to the reference genes described by Hartney *et al*. [25]. Sequences were aligned with Gap open cost 15 and Gap extension cost 0.3. Neighbor joining trees were created by performing bootstrap analysis with 1000 replicates.

## Results

### *P. putida* W15Oct28 whole genome sequence analysis and comparison with other sequenced *P. putida* strains

**Figure 1:**
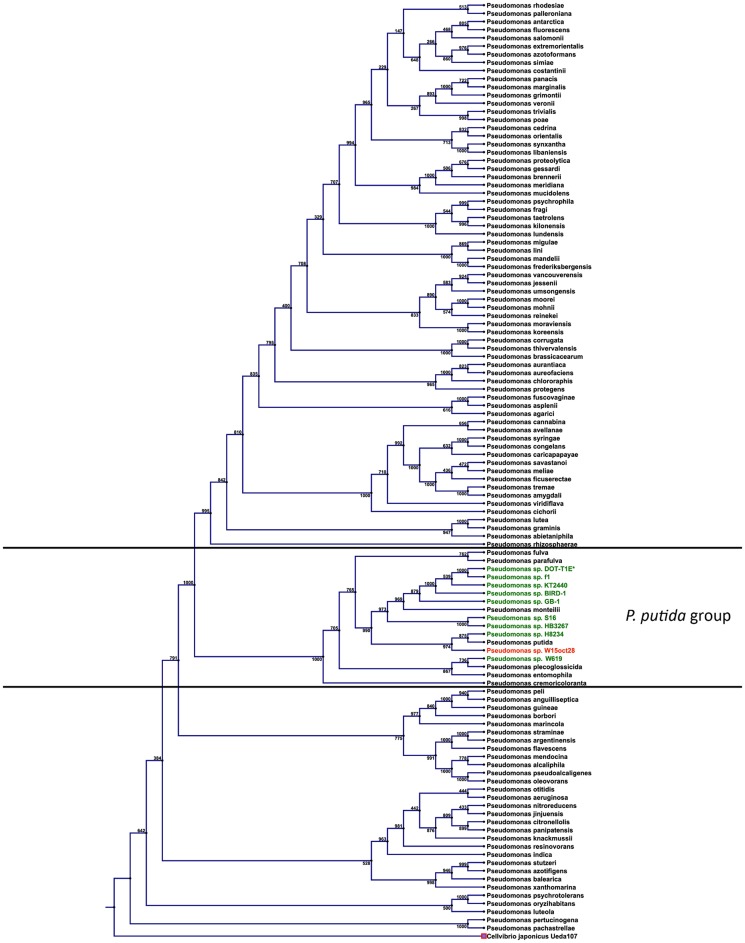
Pseudomonas phylogenetic tree. A phylogenetic tree of different Pseudomonas species based on the comparison of four different housekeeping genes sequences (16s rDNA, *gyrB*, *rpoB*, *rpoD*). The *P. putida* cluster is highlighted and the strain W15Oct28 is indicated in red. W15Oct28 closest relative is the *P. putida* type strain NBRC14164^T^.

The genome assembly resulted in 138 contigs assembled in 99 scaffolds representing the draft genome (longest scaffold size: 283,647 bp, 22 scaffolds longer that 100 kb, mean scaffold size 4,5726 bp, N50 scaffold size: 105,817 bp); the genome is 6,331,075 bps in length with average GC content of 62.8%. This Whole Genome Shotgun project has been deposited at DDBJ/EMBL/GenBank under the accession JENB00000000. The version described in this paper is version JENB01000000, biosample SAMN02644482. The genome contains 5,540 predicted coding sequences (CDS), a total of 116 RNA genes, including 6 rRNA operons (8 copies of 5S rRNAs, 6 copies of 16S rRNAs, and 6 copies of 23S rRNAs). In addition, 71 tRNA genes were identified. The W15Oct28 strain was clearly confirmed to be *Pseudomonas putida* by analysis of different housekeeping genes sequences (16s rDNA, *gyrB*, *rpoB*, *rpoD*) according to the phylogeny method described by Mulet *et al*. [10], [26] with 99%, 97%, 98%, and 99% DNA sequence identity, respectively with the type strain of *P. putida* NBRC14164^T^ (**Figure 1**). It is interesting to notice that the closest relatives of W15Oct28 are the *P. putida* type strain NBRC 14164^T^, and the recently sequenced strain H8234, a clinical isolate [27], confirming that our strain probably belongs to the species *P. putida* [28]. It appears also from the phylogenetic tree of Figure 1 that the other strains designated as *P. putida* probably represent other species. As a confirmation of this preliminary taxonomical assignment, **Table 1** shows the Average Nucleotide Identity based on BLAST (ANIb) between the genomes of all the *P. putida* group strains represented in Figure 1. The table confirms that *P. putida* W15Oct28 shares about 94% of its genome with the recently sequenced NBRC14164^T^ strain and 92% with H8234, totally in line with the previous phylogenetic assignment presented in Figure 1. The percentage of identity with other representatives of the *P. putida* group is lower, again confirming the previous multi-locus analysis-based taxonomic assignment. *P. putida* KT2440, BIRD 1, and DOT T1E form another cluster of related strains (**Table 1**). The circular map of the *P. putida* W15Oct28 genome is presented in **Figure S1**.

**Table 1:** Whole genome comparison (Average Nucleotide Identitybased on BLAST [ANIb]) between different *P. putida* group strains, including W15Oct28.

ANIb	*Pseudomonas putida* NBRC 14164	*Pseudomonas* sp. W15oct28	*Pseudomonas* sp. H8234	*Pseudomonas* sp. GB 1	*Pseudomonas* sp. KT2440	*Pseudomonas* sp. BIRD 1	*Pseudomonas* sp. F1	*Pseudomonas* sp. DOT T1E	*Pseudomonas* sp. S16	*Pseudomonas* sp. HB3267	*Pseudomonas* sp. W619	*Pseudomonas entomophila* L48	*Pseudomonas* sp. UW4	*Pseudomonas* fulva 12 X
*P. putida* NBRC 14164**^T^**	–	**94.2**	**93.3**	90.4	89.7	89.6	89.5	89.5	89.4	89.5	85.2	84.2	77.3	76.2
***Pseudomonas*** ** sp**. **W15oct28**	**94.2**	–	**92.5**	90.4	89.5	89.6	89.4	89.4	89.5	89.4	85.0	84.1	77.2	76.0
*Pseudomonas* sp. H8234	**93.1**	**92.4**	–	89.7	88.9	88.9	88.8	88.9	88.4	88.6	84.8	83.9	77.1	75.7
*Pseudomonas* sp. GB 1	90.4	90.6	89.9	–	90.3	90.4	90.5	90.4	89.4	89.7	85.1	84.2	77.2	76.0
*Pseudomonas* sp. KT2440	89.7	89.7	89.2	90.4	–	**97.2**	**96.7**	**96.9**	88.9	89.2	84.9	84.0	77.2	76.0
*Pseudomonas* sp. BIRD 1	89.7	89.8	89.2	90.6	**97.3**	–	**96.7**	**96.8**	89.1	89.2	84.6	84.1	77.3	76.1
*Pseudomonas* sp.F1	89.6	89.5	89.1	90.5	**96.7**	**96.6**	–	**98.3**	88.9	89.2	84.8	84.0	77.1	76.0
*Pseudomonas* sp. DOT T1E	89.4	89.4	89.1	90.2	**96.7**	**96.5**	**98.1**	–	88.8	88.8	84.5	84.0	76.9	75.7
*Pseudomonas* sp. S16	89.5	89.6	89.0	89.5	88.9	89.1	89.0	89.0	–	**96.9**	85.4	84.9	77.7	76.7
*Pseudomonas* sp. HB3267	89.6	89.6	89.0	89.8	89.2	89.1	89.2	89.0	**96.9**	–	85.8	84.9	77.7	76.7
*Pseudomonas* sp. W619	85.3	85.3	85.1	85.2	84.9	84.6	84.8	84.7	85.4	85.7	–	84.0	76.8	75.8
*P. entomophila* L48	84.2	84.2	84.2	84.3	84.1	84.0	84.1	84.2	84.9	85.0	83.9	–	77.8	77.1
*Pseudomonas* sp. UW4	77.2	77.2	77.1	77.2	77.2	77.1	77.1	77.0	77.5	77.6	76.8	77.5	–	75.7
*P. fulva* 12 X	76.31	76.45	76.07	76.26	76.17	76.18	76.19	76.13	76.73	76.72	75.83	77	75.94	–

### Genes corresponding to secondary metabolism pathways

#### A. Biosynthetic pathway of a new type pyoverdine and the presence of 56 TonB-dependent receptors

**Figure 2:**
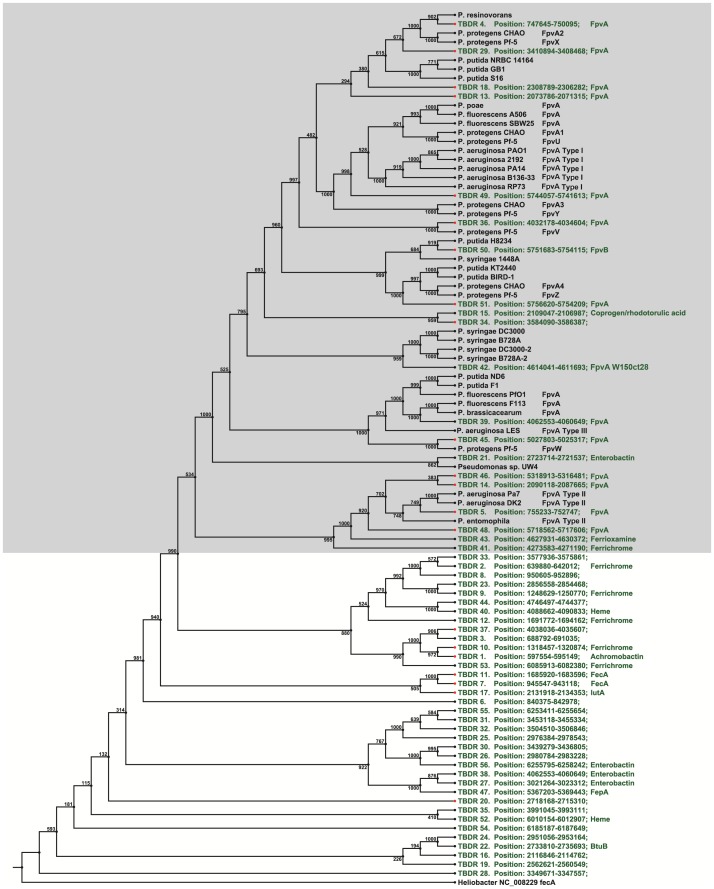
Phylogeny of TonB-dependent receptors. Neighbor joining tree based on the alignment of amino acid sequences of the 56 TonB dependent receptors (TBDR) detected in the genome of W15oct28 (green font) and a selection of known ferric-pyoverdine receptors from different sequenced Pseudomonas genomes (black font). W15oct28 TBDRs that are regulated through sigma-anti-sigma factors are indicated with a red node. Grey surface indicates part of the tree that contains all ferric-pyoverdine receptors that form a separate cluster.

In our previous work, we reported that the only form of pyoverdine produced by *P. putida* W15Oct28 contains α-keto-glutaric acid as acyl side chain, a dihydropyoverdine chromophore and a 12 amino acid peptide chain [12]. One unique aspect of this pyoverdine is the incorporation of L-homoserine in peptide chain which was only previously reported in the pyoverdine produced by *Azotobacter vinelandii* DJ [12]. The pyoverdine biosynthesis gene clusters are localized in three regions in the genome. The first pyoverdine genomic region shares high identity (99%) with other *P. putida* type strains and contains the genes which catalyze the formation of the chromophore precusor (PvdL/G/Y/H) while the last part of the cluster contains genes involved in the transport of ferripyoverdine (*fpvC*, *fpvD*, *fpvE*, *fpvF*) [29] and in pyoverdine modification (*pvdA*, *pvdQ*) [30], [31]. The second cluster of genes shares about 75% identity with other whole genome sequenced *P. putida* strains and contains the genes *pvdM*, *pvdN*, *pvdO*, *pvdP* encoding periplasmic enzymes which are involved in the final maturation of the chromophore in the periplasm [32]. The third cluster contains one thioesterase gene, the genes for four non-ribosomal peptide synthetases involved in the peptide chain synthesis (*pvdD*, *pvdI*, *pvdJ*, *pvdK*) and the *fpvA* gene corresponding to the TonB-dependent ferri-pyoverdine receptor. These last genes share less than 50% identity with the corresponding genes from other *P. putida* strains [12]. This third pyoverdine cluster was considered by IslandViewer to correspond to a genomic island (GIs), because of its lower GC content compared to the average. Details about the genes involved in W15Oct28 pyoverdine biosynthesis and uptake have been described in our previous work [12]. *Pseudomonas putida* W15Oct28 genome contains 56 genes encoding TonB-dependent outer-membrane proteins receptors, 20 of them being probably involved in ferri-pyoverdine uptake, a situation similar to what we observed in *P. fluorescens* ATCC17400 which has 55 TonB-dependent receptors, including 17 that are involved in the uptake of different ferric pyoverdines [33]. Analysis of these receptors compared to other characterized *Pseudomonas* TonB-dependent receptors, revealed the presence of homologs of FpvAI, FpvAII, FpvAIII, and FpvU/V/W/X/Y/Z already described in *P. protegens* Pf-5 [25] (**Figure 2**). Pyoverdine feeding experiment confirmed the utilization of *P. aeruginosa* type I, II and III pyoverdines and the pyoverdines produced by *P. protegens*, and *P. fluorescens* species (**Table 2**). Other receptors predicted to recognize siderophores produced by other bacteria were found as well (enterobactin, achromobactin). The W15Oct28 FpvA receptor for its own pyoverdine utilization showed the best blast hit with the FpvA of *P. syringae* (42% identity), and it clusters with the *P. syringae* FpvA receptors in the tree presented in Figure 2, confirming the unique structure of the pyoverdine we recently disclosed [12]. The list of TonB-dependent receptors is presented in **Table S2 in file S1**.

**Table 2:** Pattern of pyoverdines utilization by the *pvdL*-pyoverdine-negative mutant.

Producing strain	MW/Da	Peptide chain	Utilization	Other producing strain
*Pseudomonas* sp. W2Aug36	989	εlys-OHAsp-Ala-aThr-Ala-cOHOrn	yes	*Pseudomonas* sp. B10
*P. umsongensis* LMG 21317T	1046	Ala-Lys-Thr-Ser-AcOHOrn-cOHOrn	yes	*P. fluorescens* Ps4a
*P. aeruginosa* PAO1 (type I)	1333	Ser-Arg-Ser-FOHOrn-(Lys-FOHOrn-Thr-Thr)	yes	
*P. aeruginosa* W15Oct32 (type II)	1091	Ser-FOHOrn-Orn-Gly-aThr-Ser-cOHOrn	yes	*P. aeruginosa* Pa 27853
*P. aeruginosa* 59.20 (type III)	1173	(Ser–Dab)–FOHOrn–Gln–Gln–FOHOrn–Gly	yes	*P. aeruginosa* Pa6/(R)
*P. rhodesiae* Lille 25	1421	Ser-Lys-FOHOrn-Ser-Ser-Gly-(Lys-FOHOrn-Ser-Ser)	yes	
*P. protegens* Pf-5	1287	Asp-FOHOrn-Lys-(Thr-Ala-Ala-FOHOrn-Lys)	yes	*P. protegens* CHA0
*P. lurida* LMG 21995T	1364	Ser–Ser–FOHOrn–Ser–Ser–(Lys–FOHOrn–Lys–Ser)	yes	*P.fluorescens* 95–275
*P. salomonii* LMG 22120T	1263	Ser–Orn–FOHOrn–Ser–Ser–(Lys–FOHOrn–Ser)	yes	*Pseudomonas* sp. 96–318
*P. brenneri* LMG 23068T	1187	Ser–Dab–Gly–Ser–OHAsp–Ala–Gly–Ala–Gly–cOHOrn	yes	*P.fluorescens* PflW
*P. aureofciens*	1277	Ser-AOHOrn-Gly-aThr-Thr-Gln-Gly-Ser-cOHOrn	yes	*P.aureofaciens* P. au
*P. citronellolis* LMG 18378T		structure unknown	yes	
*Pseudomonas* sp. W15Feb38	1046	Ser-AOHOrn-Ala-Gly-aThr-Ala-cOHOrn	yes	*P. fluorescens* PL7
*Pseudomonas* sp. W2Feb31B	1246	structure unknown	yes	
*Pseudomonas* sp. W2Jun14	1159	structure unknown	yes	
*P. chlororaphis* W2Apr9		structure unknown	yes	
*P. putida* F1	1370	Asp-OHbutOHOrn-Dab-Thr-Gly-Ser-Ser-OHAsp-Thr	yes (slightly)	*P. putida* PutC
*P. fluorescens* ATCC 17400	1299	Ala–Lys–Gly–Gly–OHAsp–(Gln–Dab)–Ser–Ala–cOHOrn	yes (slightly)	
*P. fluorescens* ATCC 17926	1159	structure unknown	yes (slightly)	
*P. syringae* B728a	1123	εLys–OH Asp–Thr–(Thr–Ser-OH Asp–Ser)	yes (slightly)	
*P. brassicacearum* LMG 21623T	1150	Ser-AOHOrn-Ala-Gly-(Ser-Ser-OHAsp-Thr)	yes (slightly)	*P. fluorescens* PL9
*P. putida* KT2440	1072	Asp–Orn–(OHAsp–Dab)–Gly–Ser–cOHOrn	No	*P. putida* G4R and BIRD-1
*P. putida* L1	1349	Asp-εLys-OHAsp-Ser-aThr-Ala-Thr-Lys-cOHOrn	No	*P. putida* GB-1 and WCS358
*P. fluorescens* BTP2	1049	Ser–Val–OHAsp–Gly–Thr–Ser–cOHOrn	No	
*P. fluorescens* Pf0-1	1381	Ala–AcOHOrn–Orn–Ser–Ser–Ser–Arg–OHAsp–Thr	No	
*P. entomophila* L48	1314	Ala-xxx-OHHis-Asp-Gly-Gly-Ser-Thr-Ser-cOHOrn	No	
*P. vancouverensis* LMG 20222T		structure unknown	No	
*P. asplenii* LMG 21749T		structure unknown	No	
*P. koreensis* MFY71		structure unknown	No	

#### B. Gene clusters for the biosynthesis of putisolvins

**Figure 3:**
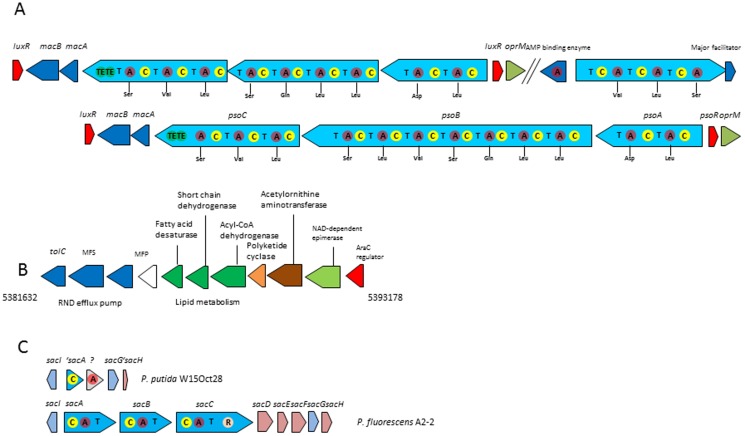
Genes involved in the production of secondary metabolites. A. Genes involved in the production of putisolvins in strain W15Oct28 (top) and strain *P. putida* PCL1145 (bottom): *psoA*, *psoB*, and *psoC* are the genes encoding NRPS enzymes with their condensation (C), adenylation (A) domains showing the predicted activated amino acid, and the T domain for the thioester attachment of the activated amino acid. The two thioesterase domains responsible for the detachment of the completed peptide at the end of *psoC* are also indicated (TE). The *macA* and *macB* genes correspond to a transporter, the *oprM* gene coding for an efflux system porin, and the two orphan *luxR* genes are shown in red. The amino acids predicted to be activated by the different A domains by the antiSMASH analysis are indicated without mentioning whether they are in the D- or L- form. B. The ten genes cluster possibly involved in the biosynthesis of a secondary metabolite. The cluster is preceded by a gene encoding an AraC regulator. See the text for details. C. The incomplete safracin gene cluster of W15Oct28 compared to the complete safracin gene cluster of *P. fluorescens* A2-2.

The culture supernatant of *P. putida* W15Oct28 has a very low surface tension, which is likely to be the result of biosurfactant production, such as putisolvins. Putisolvins are lipopeptides biosurfactants produced by *P. putida* strains, such as *P. putida* PCL1445 and 267 [34]–[39]. Putisolvins were reported to increase motility and prevent biofilm formation of the producing strain, and can cause existing biofilms disruption. The structure of putisolvin I is C6-Leu-Glu-Leu-Ile-Gln-Ser-Val-Ile-c(Ser-Leu-Val-Ser), and its molecular weight is 1,379 Da. Putisolvin II has 14 Da more in mass due to the replacement of a valine to leucine or iso-leucine on the 11th amino acid position. **Figure 3a** is showing the organization of the putisolvin genes cluster of W15 Oct28 and *P. putida* PCL1445. Both genes clusters are highly similar and antiSMASH predicted the same amino acids to be activated by the different adenylation (A) domains. Although the three NRPS genes *psoABC* are in one cluster in *P. putida* PCL1145, the antiSMASH analysis found two different clusters for *P. putida* W15Oct28, one containing three NRPS genes while the remaining NRPS gene is found in another cluster. In both instances, the NRPS genes are flanked on one side by a LuxR regulator gene and by *macA* and *macB* genes encoding a transporter. On the other side of the PCL1145 cluster and in the second cluster of *P. putida* W15 Oct28, there is another *luxR* gene (*psoR*) and a gene coding for an outer membrane efflux protein (OprM). Although the amino acid sequences of the three non-ribosomal peptide synthetases (PsoA/B/C) only share an average of 80% identity between W15Oct28 and the sequences reported in PCL1445 (access no. DQ151887.2), only few differences in the signature residues of 3 adenylation domains for substrate recognition and activation in PsoB were found: the second A domain for leucine activation (W15Oct28: DAWSLGNV; PCL1445: DAWFLGNV), the fifth A domain for valine activation (W15Oct28: DALWMGGT; PCL1445: DALWIGGT), and the seventh A domain for serine activation (W15Oct28: DVWHXXXX; PCL1445: DVWHLSLV). However, the antiSMASH software analysis predicted the same amino acids to be activated by the different putisolvin NRPS domains in PCL1145 and W15Oct28 (**Figure 3a**). The biosurfactants produced by *P. putida* W15Oct28 were also characterized as putisolvins by high-resolution and CID mass spectrometry (both molecular weight and amino acid sequence are identical to the reported data). In the LC/MS results presented in **Figure S2a and b** for putisolvins I and II, respectively, reveal one product with a mass of 1,380 (putisolvin I) and one with a mass of 1,394 (1,380 plus 14 Da for putisolvin II). These values are very close to those found for the two *P. putida* PCL1445 putisolvins I and II (1,379 and 1,393, respectively).

The *ppuI*-*rsaL*-*ppuR* quorum sensing system which regulates putisolvins production in PCL1445 [36] is apparently missing in the genome of W15Oct28.

#### C. Production of a broad-spectrum antimicrobial molecule

**Figure 4:**
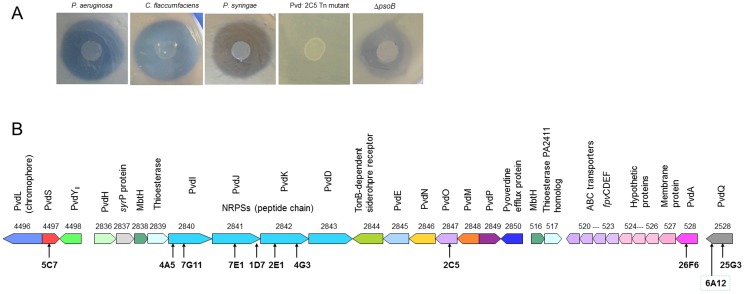
Antagonistic activity and role of pyoverdine in the antagonism. A. Antagonistic activity of the *P. putida* W15Oct28 strain against *Pseudomonas aeruginosa*, *Curtobacterium flaccumfaciens*, and *Pseudomonas syringae*. The pyoverdine-negative *pvdO* 2C5 transposon mutant has lost its antagonism against *P. aeruginosa* while the Δ*psoB* putisolvin-negative mutant keeps a reduced, but still visible level of antagonism. B. The pyoverdine genes clusters: the arrows indicate the places of transposon insertions causing the loss of pyoverdine production and of the antagonism.

One of the most interesting features of *P. putida* W15Oct28 is its strong antagonistic activity against several bacterial human pathogens (*P. aeruginosa*, *S. aureus, S. epidermidis*), the entomopathogenic *P. entomophila*, several plant pathogens (different pathovars of *P. syringae*, *Xanthomonas translucens* pv. *cerealis* LMG679, *Curtobacterium flaccumfaciens* [Gram-positive], and the yeast *Saccharomyces cerevisiae*) (**Table 3** **and** **Figure 4a**). The production of the antimicrobial molecules could be detected after growing the strain W15Oct28 on M9 minimal medium supplemented with different carbon sources (glucose, citric acid or glycerol). Interestingly, the production of the antimicrobial molecule(s) was only detected when the cells were grown in media low in iron and organic nitrogen sources. The production of the antimicrobial compound(s) was detected both on solid and liquid aerated media (160 rpm, at 26–28°C). When screening a transposon mutant library for antagonism-negative mutants of W15Oct28, the majority of them turned out to be pyoverdine null mutants with transposon insertions in different pyoverdine biosynthesis genes (**Figure 4b**), which is in agreement with the fact that the antagonistic activity was lost in medium supplemented with iron, due to the suppression of pyoverdine production. The production of the antimicrobial molecule(s) was not observed when the cells were grown the in iron-poor and organic nitrogen sources-rich casamino acid medium, which allows high levels of pyoverdine production, suggesting that the antimicrobial compound is not pyoverdine. We also constructed an in-frame deletion mutant in the *pvdL* gene encoding an NRPS gene for the pyoverdine chromophore precursor synthesis [40]. As expected, the *pvdL* mutant lost its antagonism, confirming the link between pyoverdine production and the antagonistic activity. We also observed that when the CbrA/B two component regulation system [41] was deactivated, pyoverdine production was unchanged but no antimicrobial activity could be detected. These results suggest that the production of the antibiotic compound(s) by *P. putida* W15Oct28 is somehow linked to pyoverdine production, but is not due to pyoverdine itself. Indeed, purified pyoverdine only showed some limited growth inhibitory activity against gram-positive bacteria, probably as a result of iron deprivation (results not shown). To rule out the possibility that the antagonism could be due, totally or partially, to the production of putisolvins, we constructed a mutant with an in- frame partial deletion in the *psoB* homologue of W15Oct28 and tested it for its antagonistic activity. This mutant still maintained an inhibitory activity, albeit slightly reduced, excluding the involvement of the sole putisolvins in the antimicrobial activity of W15Oct28.

**Table 3:** Antagonistic activity of *P. putida* W15Oct28 against different microorganisms.

Bioassay indicator strain	Activity
**Gram-positive bacteria**	
*Staphylococcus aureus* NCTC 8325	+
*Staphylococcus aureus* ATCC 29213	+
Methicillin-resistant *S. aureus* B6	+
Methicillin-resistant *S. aureus* D6	+
*Staphylococcus epidermidis* RP62A	+
*Micrococcus luteus* ATCC 4698	+
*Curtobacterium flaccumfaciens*	+
**Gram-negative bacteria**	
*Pseudomonas syringae* pv. tomato DC3000	+
*Pseudomonas syringae* pv. syringae B301D	+
*Pseudomonas syringae* pv syringae B728a	+
*Pseudomonas entomophila* L48	+
*Pseudomonas aeruginosa* PAO1	+
*Pseudomonas aeruginosa* PA14	+
*Xanthomonas translucens* pv. cerealis LMG679	+
*Pseudomonas aeruginosa* PA7	−
*Pseudomonas putida* KT2440	−
*Pseudomonas protegens* Pf-5	−
*Escherichia coli* MG1655	−
*Klebsiella pneumoniae* 8401	−
*Klebsiella pneumoniae* 8410	−
*Salmonella enterica* sp. Typhimurium X3000	−
*Salmonella enteritidis* 76Sa88	−
**Eukaryotic microorganisms**	
*Pythium ultimum*	−
*Saccharomyces cerevisiae*	+

**Figure 5:**
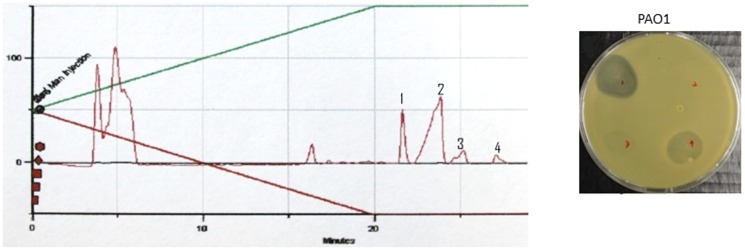
Fractionation of the antagonistic activity by HPLC. HPCL fractionation of a crude extract from a culture supernatant of *P. putida* W15Oct28 grown in M9-glucose medium: A, chromatography of crude extraction of W15Oct28 cultured in M9 minimal medium for 48 hours; B, chromatography of crude extraction of W15Oct28 cultured in M9 minimal medium for 48 hours plus 5 days stay at 6°C. The yield of fraction 1 and 4 were increased with longer time of cultivation. Fractions 1 to 4 were spotted on an agar plate inoculated with *P. aeruginosa* PAO1 (upper left, fraction 1, upper right, fraction 2, lower left, fraction 3, lower right, fraction 4). The green line represents the acetonitrile gradient.

Two antimicrobial molecules fractions, termed fraction 1 and 4, were obtained from 18 L of culture in M9 minimal medium with yields of 9 mg and 6 mg, respectively (**Figure 5**). Antimicrobial molecule fraction 1 is a colorless oily like compound while the second antimicrobial molecule present in fraction 4 is a white powder. These two molecules are both highly hydrophobic and eluted from the HPCL column only at 100% acetonitrile. Attempts to determine the mass of the molecule or to determine its structure by ^1^H or ^13^C NMR were however unsuccessful.

#### D. Other gene clusters for putative secondary metabolites

Given the fact that there is no characteristic polyketide synthase gene cluster in the genome of W15Oct28, we decided to use the antiSMASH algorithm [16], [17] to search for putative biosynthetic gene clusters containing genes corresponding to fatty acid desaturase and PKS tailoring enzymes, in an approach similar to the one used to solve the puzzle of the tunicamycin biosynthetic gene cluster [42]. One cluster of ten genes was selected by antiSMASH for its potential involvement in secondary metabolism, which is also present in the recently sequenced *Pseudomonas* sp. HYS [43]. This gene cluster presented in **Figure 3b** is preceded by a gene encoding an AraC transcription regulator, followed by genes coding for a NAD-dependent epimerase similar to the *mupV* gene from *P. fluorescens* NCIMB10586 strain involved in the synthesis of the polyketide antibiotic mupirocin [44], an acetylornithine aminotransferase that can catalyze the reversible transfer of an acetyl-group from a basic (ornithine) to an acidic (glutamate) amino acid [45], a PKS cyclase, an isobutylamine *N*-hydroxylase similar to VlmH involved in valanimycin biosynthesis [46], a FabG like short chain dehydrogenase, a fatty acid desaturase, an hypothetical protein and three proteins forming an RND family efflux transporter (MFP subunit, EmrB/Qac drug resistant transporter, and outer membrane lipoprotein of RND efflux system). However, attempts to inactivate genes in this cluster have so far failed and its involvement in the production of a bioactive molecule remains hypothetical.

Safracins were reported to be produced by two strains of *P. fluorescens*, A2-2 and SC 12695 [47]. The biosynthesis of this antitumor compound drew attention due to the fact that safracin B can be used as an intermediate for the synthesis of ecteinascidin, which is a potent anti-tumor agent [48]. The biosynthesis gene cluster of safracin in *P. fluorescens* A2-2 contains three NRPSs (SacA, SacB, SacC), three precursor biosynthetic enzymes (SacD, SacF, SacG), two tailoring enzymes (SacI, SacJ), one resistance protein (SacH) and one protein with unknown function (SacE) [47]. In the genome of *P. putida* W15Oct28, we found three genes corresponding to the beginning of the cluster (*sacJ*, *sacI*, *sacA*) (**Figure 3C**). SacA is a NRPS, but in W15Oct28 it is truncated since it only contains one condensation domain. A second short ORF containing only one adenylation domain follows, which shows no similarity with any of the genes in the safracin cluster in *P. fluorescens* A2-2. The partial cluster ends with *sacG* and *sacH* while the part from the thioesterase domain of *sacA* till the end of *sacF* is missing (**Figure 3C**).

### Secretion systems: first evidence for a Type IV secretion system in a pseudomonad

**Figure 6:**
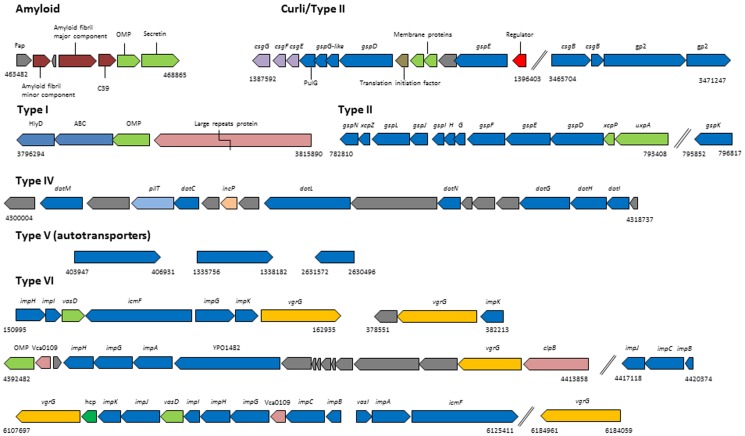
Secretion systems. Gene clusters coding for components of different secretion systems: amyloid, curli, type I, type II, type IV, type V (autotransporters) and type VI secretion systems. The genes in yellow for type VI secretion represent the different VgrG effector proteins.

*P. putida* W15Oct28 genome contains genes for different secretion systems, including a Fap amyloid fiber secretion system, a curli production system, one type I, one type II, one type II/IV secretion system, as well as one putative type IVb secretion system, three autotransporter genes (type V) and several genes for a type VI secretion system (**Figure 6**). There is however no system for type III secretion in this strain. Most of those secretion systems are conserved in other *P. putida* group species, with the exception of the Dot/Icm type IVb secretion system, although a highly similar gene cluster is present in the genome of *P. putida* BIRD-1 (PPUBIRD1_4462-4506) [49]. The new putative type IV secretion system is most similar to the T4SSb from the pathogenic Gram-negative bacterium *Legionella pneumophila* [50], which is characterized by the presence of several conjugative transfer proteins, such as TraA, IncP type conjugative transfer protein TrbN, and IncI/plasmid conjugation transfer protein TraN. Although this is the first description of the presence of genes encoding the components of Type IV secretion system in *Pseudomonas*, we do not know at this stage if it is functional. Another interesting observation is the presence in the genome of five different VgrG type VI effector proteins.

### Evidence for the presence of pyocins genes

Beside the production of antimicrobial compounds synthesized by secondary metabolism which are often characterized by a broad inhibitory spectrum, bacteria also produce antimicrobial peptides or proteins termed bacteriocins to kill their close relatives (usually within the same species) [51]–[53]. The best studied bacteriocins are those produced by *E. coli*, termed colicins, and the pyocins produced by *P. aeruginosa* and other pseudomonads [51], [54]. Pyocins production genes are found in the genome of strain W15Oct28, including a CvpA colicin V production protein and the CreA colicin E2 immunity protein which are conserved in different *P. putida* genomes. There is also the presence of an S-type pyocin gene encoding a protein with translocation domain and killing domain similar to the one in strain KT2440 while the immunity gene is similar to the one in strain GB-1 (results not shown). The killing domain contains a HNH DNA endonuclease motif, which shares similarity with the S1/S2 type pyocins which are present in different *P. aeruginosa* strains [51]. Besides its presence in strain KT2440 as described by Parret and De Mot [54], this pyocin killing domain could also be found in *P. putida* strains ND6 and S13.12. The immunity gene adjacent to the gene of killing domain shows similarity to the immunity gene of the *E. coli* E7/8 colicin.

## Discussion

The *Pseudomonas* genus definition has a long history, which first started with phenotypic characterizations and metabolic pathway profiling, later followed by 16s rDNA phylogeny, which resulted in the exclusion of some species from the true pseudomonads which cluster in the rRNA group I [55], [56]. Now a new chapter is opening based on whole genome phylogeny as exemplified in this and other studies [57], [58]. Although the species *P. putida* and *P. fluorescens* represent a large group of *Pseudomonas* species adapted to diverse environmental niches, the number of sequenced genomes is still low compared to the large number of genomes from the well-studied pathogenic species *P. aeruginosa* [49]. Next generation sequencing methods which became available in the last few years promoted a surge in taxonomic study based on whole genome comparison. Recently, an increasing number of genomes of strains belonging to the *P. putida* group have been reported, providing an opportunity to better understand how this species can adapt to different environmental niches using a combination of bioinformatics and experimental approaches. By combining a multi locus sequencing approach (MLSA) and whole genome comparison, we could confirm that our strain W15Oct28 is indeed a true *P. putida* since its closest relative is the *P. putida* type strain NBRC 14164^T^ [28]. We believe that the strain W15Oct28 described in this study represents a good example of how diverse *P. putida* strains can be. Strain W15Oct28 was isolated from the river Woluwe in Brussels [11] and it attracted our attention because of its antagonism towards *Staphylococcus aureus* and *P. aeruginosa*. This strain produces a pyoverdine with a mass as 1,624 Da as the only siderophore [12]. The very low surface tension of cultures supernatants also suggested the production of biosurfactant(s). We could indeed identify that W15Oct28 produced two putisolvins by mass spectrometry, and the amino acid sequence of putisolvins deduced from the analysis of NRPS genes showed that they shared 80% identity with the *P. putida* PCL1445 putisolvins [36], [37].

The most striking characteristic of W15Oct28 is its strong antagonistic activity against several bacterial pathogens, which is due to the production of a highly hydrophobic antimicrobial molecule, the structure of which could not yet be determined, due to the difficulty to ionize the molecule. An intriguing observation was that a majority of transposon mutants which were unable to produce the compound had insertions in genes involved in the biosynthesis of pyoverdine, despite the fact that pyoverdine itself showed no antimicrobial activity. One possible explanation is that the proteins involved in the production of the antibiotic compound share the same “siderosome” platform as the pyoverdine biosynthetic machinery recently described in *P. aeruginosa* [59]–[61]. Another surprising result was the discovery in the genome of an incomplete gene cluster for the biosynthesis of the antibiotic safracin, which is produced by a *P. fluorescens* strain [47]. We hypothesize that this entire gene cluster was originally present in the genome and became later on deactivated by deletion. Besides its particular secondary metabolism, W15Oct28 possesses other traits which are not shared by other *P. putida* group species. W15Oct28 was found to contain a putative type IVb secretion system, which could only be found in the genome of *Pseudomonas sp*. BIRD-1, but not in other known *Pseudomonas* genomes. Strain W15Oct28 also contains a fatty acid biosynthesis gene cluster which is only present in two newly whole genome sequenced strains *P. putida* NBRC 14164^T^ and H8234 [27], [28], which are the closest relatives of W15Oct28. The W15Oct28 strain has also 56 TonB-dependent receptors and most of them have highly conserved homologs in other *P. putida* strains, especially GB-1 and NBRC 14164, which makes this strain one of the *Pseudomonas* having the largest repertoire of TonB-dependent receptors, comparable with *P. fluorescens* ATCC17400 which has 55 receptor genes [33]. Cross feeding results show that *P. putida* W15Oct28 is able to utilize pyoverdines produced by *P. aeruginosa* and *P. fluorescens* strains while it shows a limited capacity to use pyoverdines produced by other *P. putida*, in agreement with the lack of known *P. putida* ferric-pyoverdine receptors in W15Oct28 genome (results not shown). On the other hand, we assume that the pyoverdine produced by W15Oct28 is not commonly found in *Pseudomonas* sp. strains since its corresponding receptor has no homolog with an identity above 50% in Genbank so far. Results of the antagonism tests show that this strain has a promising inhibitory activity against both Gram-positive and Gram-negative phytopathogens, especially against different pathovars of *P. syringae*. In contrast, strain W15Oct28 shows no antagonism against non-pathogenic *Pseudomonas* strains. It remains to be seen why the production of this antimicrobial compound(s) is dependent on the pyoverdine biosynthetic machinery, and to elucidate the structure of the bioactive compound(s) produced by this particular strain of *P. putida*.

## Supporting Information

Figure S1 **Circular representation of the** ***P. putida*** **W15Oct28 genome.** The green and purple inner circle represents the GC skew while the black circle represents the GC content. The blue, green and purple circles represent the ORFs the products of which show a BLAST hit with *P. putida* NBRC 14164, BIRD-1 and GB1, respectively. The two external red circles represent the different ORFs in the bottom and the top strand, respectively. The genomic islands detected via the island finder (see text for details) are indicated by blue triangles. The yellow arrow shows the location in the genome where the ten genes cluster possibly involved in the biosynthesis and secretion of the antimicrobial compound is found. The partial safracin gene cluster is indicated by a purple triangle and the other clusters for pyoverdine biosynthesis, fatty acid synthesis, and type IV secretion system are indicated by colored arcs.
(TIF)

Figure S2 **LC/MS Mass spectra of extracted putisolvin I (A) and II (B) of** ***P. putida*** **W15Oct28.** The arrows indicate the masses. See text in the results section for details.
(TIF)

File S1 **Table S1**. List of strains and plasmids used in this study. **Table S2**. List of TonB-dependent receptors.
(DOCX)
