# Supplementary figures and images for: Draft Genome Sequence Analysis of a Pseudomonas putida W15Oct28 Strain with Antagonistic Activity to Gram-Positive and Pseudomonas sp. Pathogens
Source: PLoS One. 2014 Nov 4;9(11):e110038. doi: 10.1371/journal.pone.0110038 (PMC4219678; doi:10.1371/journal.pone.0110038)

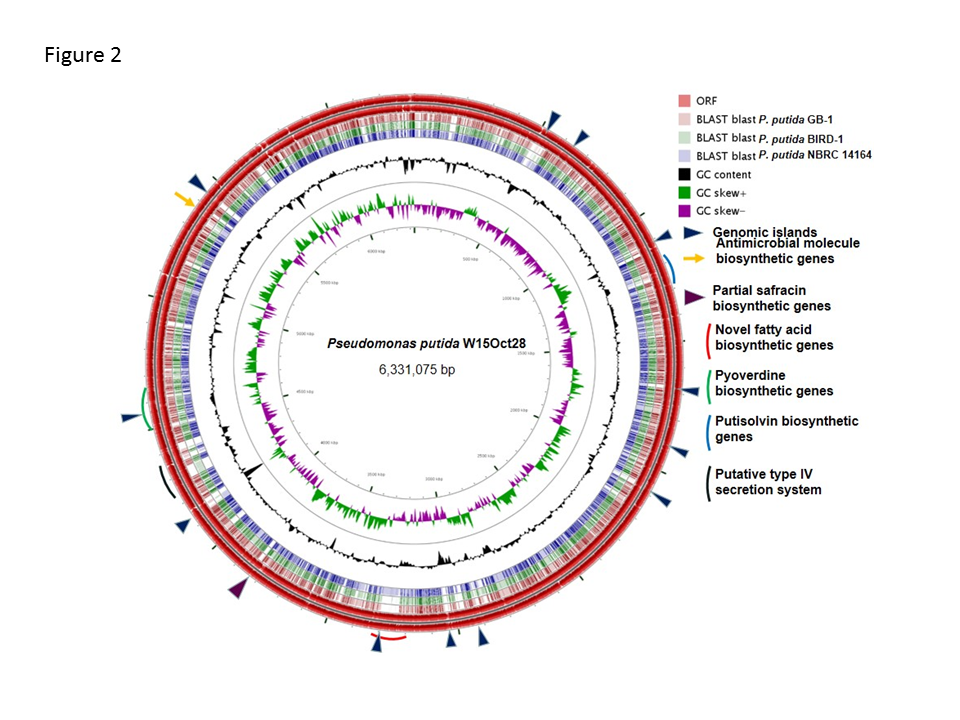

Supplement: Figure S1 — Circular representation of the P. putida W15Oct28 genome. The green and purple inner circle represents the GC skew while the black circle represents the GC content. The blue, green and purple circles represent the ORFs the products of which show a BLAST hit with P. putida NBRC 14164, BIRD-1 and GB1, respectively. The two external red circles represent the different ORFs in the bottom and the top strand, respectively. The genomic islands detected via the island finder (see text for details) are indicated by blue triangles. The yellow arrow shows the location in the genome where the ten genes cluster possibly involved in the biosynthesis and secretion of the antimicrobial compound is found. The partial safracin gene cluster is indicated by a purple triangle and the other clusters for pyoverdine biosynthesis, fatty acid synthesis, and type IV secretion system are indicated by colored arcs. (TIF) [file pone.0110038.s001.tif]

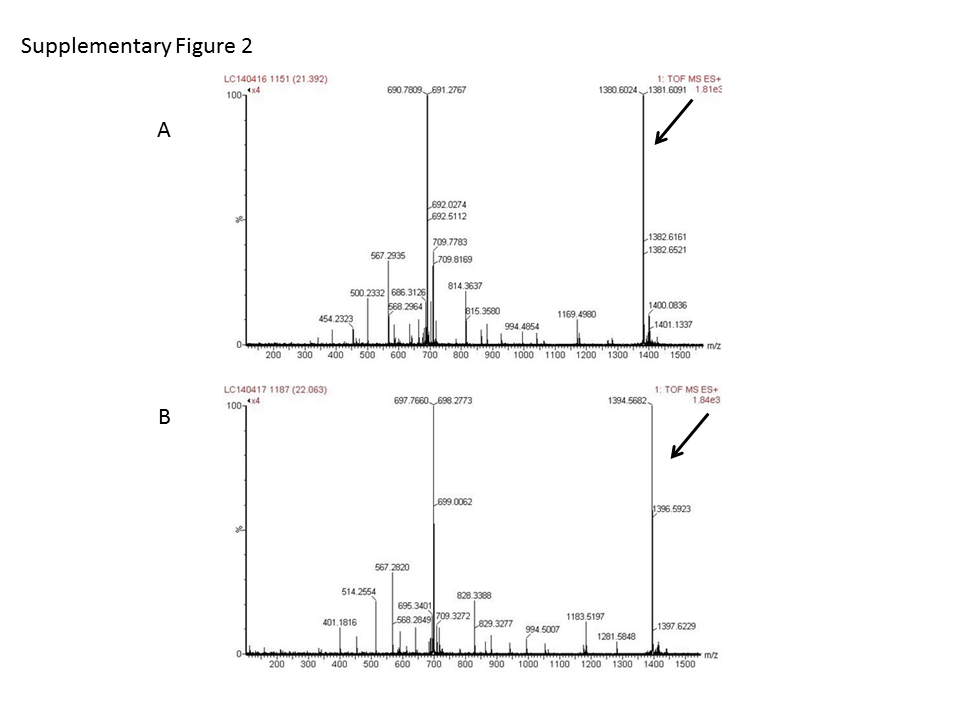

Supplement: Figure S2 — LC/MS Mass spectra of extracted putisolvin I (A) and II (B) of P. putida W15Oct28. The arrows indicate the masses. See text in the results section for details. (TIF) [file pone.0110038.s002.tif]
