# Supplementary material for: Draft Genome Sequence Analysis of a Pseudomonas putida W15Oct28 Strain with Antagonistic Activity to Gram-Positive and Pseudomonas sp. Pathogens
Source: PLoS One. 2014 Nov 4;9(11):e110038. doi: 10.1371/journal.pone.0110038 (PMC4219678; doi:10.1371/journal.pone.0110038)
Supplement: File S1 — Table S1. List of strains and plasmids used in this study. Table S2. List of TonB-dependent receptors. (DOCX) [file pone.0110038.s003.docx]

**Table S1:** Strains, plasmids and primers used in this study

| **Strain/plasmid** | **Genotype/ relevant characters** | **Reference** |
| --- | --- | --- |
| *P. putida* W15Oct28 | Wildtype, river isolate | [1] |
| *P. putida* 2C5 | pyoverdine null, inhibition abolished/ Tn5:: *pvdO* | This study |
| *P. putida* 4G3 | pyoverdine null, inhibition abolished/ Tn5:: *pvdK* | This study |
| *P. putida* 7G11 | pyoverdine null, inhibition abolished/ Tn5:: *pvdI* | This study |
| *P. putida* Δ*pvd*L | pyoverdine null, inhibition abolished/ partial *pvdL* gene is deleted | This study |
| *P. putida* ΔAMC | NRPS and methylation domain in safracin partial gene cluster deleted | This study |
| *P. putida* Δ*pso*B | putisolvin null/ partial *psoB* gene is deleted | This study |
| *E. coli* DH5α | d*lacZ* Delta M15 Delta(*lacZYA*-*argF*) U169 *recA*1 *endA*1 *hsdR*17(rK-mK+) *supE*44 *thi*-1 *gyrA*96 *relA*1 | [2] |
| *E. coli* WM3064 | conjugation doner/thrB1004 pro thi rpsL hsdS lacZΔM15 RP4-1360 Δ(*araBAD*)567 Δ*dapA*1341::[*erm pir*] | W. Metcalf |
| *E. coli* WM3064 :: pMQ30 | *ori*ColE1, *CEN6*, *ARSH4*, *aac*C1, *lac*Zα, *ori*T, *sac*B | [3] |
| *E. coli* SM10 :: pTnModOTc | RP4 *ori*T Tn5 Tc^r^ | [4] |
| *E. coli* CM404:: pRK2013 | helper plasmid | [5] |
| *S. cerevisiae* InvSc1 | Diploid *ura3-52*/*ura3-52* | Invitrogen |
|  |  |  |
| **Primers** | **Sequence: 5'-3'** | **Reference** |
| PITC-F | CGA TGA GCG CAT TGT TAG AT | [4] |
| PITC-R | TCG GCC AGC TCG TCG GTG TA | [4] |
| pvdLUP_F | GGAATTGTGAGCGGATAACAATTTCACACAGGAAACAGCTG TTGTGGATGATYTGCAGCAT | This study |
| pvdLUP_R | CAGCAYCAGACCATCCAGAC CAGTTCGACCAGCAGTTCGA | This study |
| pvdLDOWN_F | TCGAACTGCTGGTCGAACTG GTCTGGATGGTCTGRTGCTG | This study |
| pvdLDOWN_R | CCAGGCAAATTCTGTTTTATCAGACCGCTTCTGCGTTCTGAT CGCTGATGGGCAACGGCAAG | This study |
| O28AMCUp-F | GGAATTGTGAGCGGATAACAATTTCACACAGGAAACAGCTG GGCTAACGGGCAGTAGAGGA | This study |
| O28AMCUp-R | CGCTCGCAGTGACGTTCCAT CCAGTGAGCGTACCCAACTC | This study |
| O28AMCDown-F | GAGTTGGGTACGCTCACTGG ATGGAACGTCACTGCGAGCG | This study |
| O28AMCDown-R | CCAGGCAAATTCTGTTTTATCAGACCGCTTCTGCGTTCTGAT TGCTTGGTCCGCACCTGTGA | This study |
| O28psoBUp-F | GGAATTGTGAGCGGATAACAATTTCACACAGGAAACAGCTG GCTCTTTCCACCCGTGCGAT | This study |
| O28psoBUp-R | AGGTGCGATGTGACGGGTGG CAGCACGCTACCGAACACCA | This study |
| O28psoBDown-F | TGGTGTTCGGTAGCGTGCTG CCACCCGTCACATCGCACCT | This study |
| O28psoBDown-R | CCAGGCAAATTCTGTTTTATCAGACCGCTTCTGCGTTCTGAT CGCAAGGAGGCGTTCGGTAG | This study |

**Table S2:** List of TonB-dependent receptors

1. Pirnay JP, Matthijs S, Colak H, Chablain P, Bilocq F, et al. (2005) Global *Pseudomonas aeruginosa* biodiversity as reflected in a Belgian river. Environ Microbiol 7: 969-980.

2. Hanahan D (1983) Studies on transformation of *Escherichia coli* with plasmids. J Mol Biol 166: 557-580.

3. Shanks RM, Caiazza NC, Hinsa SM, Toutain CM, O'Toole GA (2006) *Saccharomyces cerevisiae*-based molecular tool kit for manipulation of genes from gram-negative bacteria. Appl Environ Microbiol 72: 5027-5036.

4. Dennis JJ, Zylstra GJ (1998) Plasposons: modular self-cloning minitransposon derivatives for rapid genetic analysis of gram-negative bacterial genomes. Appl Environ Microbiol 64: 2710-2715.

5. Boyer HW, Roulland-Dussoix D (1969) A complementation analysis of the restriction and modification of DNA in *Escherichia coli*. J Mol Biol 41: 459-472.

**Figure S1:**

Circular representation of the *P. putida* W15Oct28 genome. The green and purple inner circle represents the GC skew while the balck circle represents the GC content. The blue, green and purple circles represent the ORFs the products of which show a BLAST hit with *P. putida* NBRC 14164, BIRD-1 and GB1, respectively. The two external red circles represent the different ORFs in the bottom and the top strand, respectively. The genomic islands detected via the island finder (see text for details) are indicated by blue triangles. The yellow arrow shows the location in the genome where the ten genes cluster possibly involved in the biosynthesis and secretion of the antimicrobial compound is found. The partial safracin gene cluster is indicated by a purple triangle and the other clusters for pyoverdine biosynthesis, fatty acid synthesis, and type IV secretion system are indicated by colored arcs.

**Figure S2:**

LC/MS Mass spectra of extracted putisolvin I (A) and II (B) of *P. putida* W15Oct28. The arrows indicate the masses. See text in the results section for details.
